# P-1038. Assessment of Knowledge, Factors to Adherence, and Barriers to the Implementation of the Ventilator-Acquired Pneumonia (VAP) Bundles of Care of Healthcare Providers in the Intensive Care Unit (ICU) of a Tertiary Public Hospital in the Bicol Region, Philippines

**DOI:** 10.1093/ofid/ofaf695.1233

**Published:** 2026-01-11

**Authors:** Joy Karen S Damasig

**Affiliations:** Bicol Medical Center, Naga City, Camarines Sur, Philippines

## Abstract

**Background:**

Ventilator-associated pneumonia is a significant healthcare-associated infection that occurs 48-72 hours after endotracheal intubation, leading to increased mortality, prolonged ICU stays, and higher healthcare costs. This accounts for 20% of such infections with a mortality rate of 25-50%. In the Philippines, the estimated incidence of VAP ranges from 10% to 65%, with case fatality rates exceeding 20%. To address this issue, Ventilator Care Bundles (VCBs) have been developed based on evidence-based guidelines.

**Figure:**
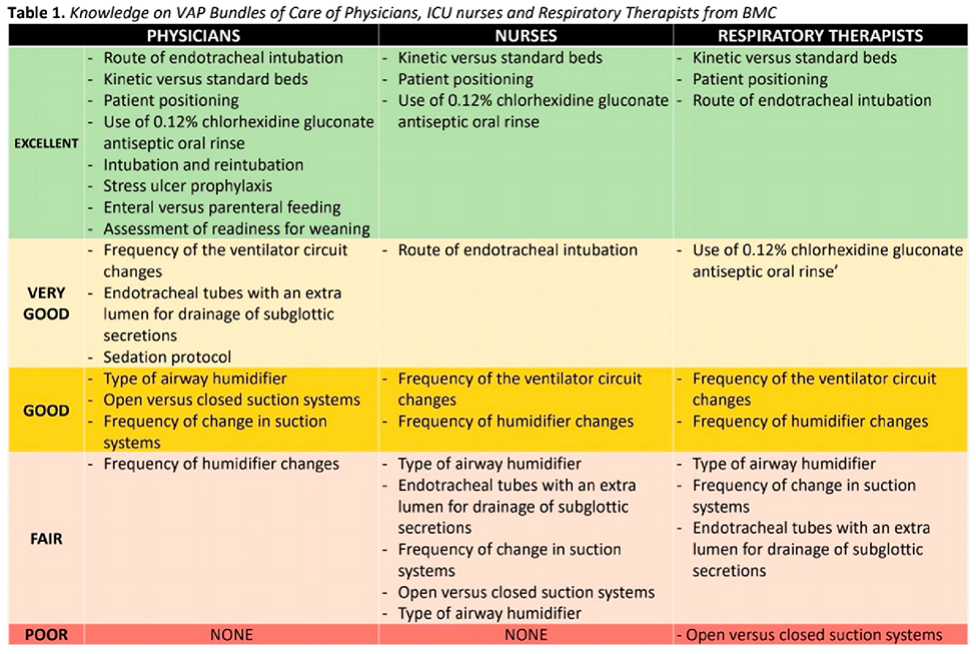
Knowledge on VAP Bundles of Care of Physicians, ICU nurses and Respiratory Therapists from BMC The results revealed varying levels of knowledge across different healthcare professional groups. Physicians generally demonstrated the highest level of knowledge, followed by respiratory therapists and nurses. All groups showed excellent knowledge on patient positioning and kinetic versus standard beds. However, knowledge gaps were identified in areas such as suction systems and airway humidifiers.

**Figure:**
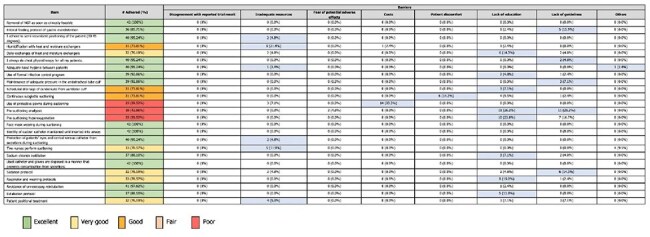
Self-reported adherence and barriers to Implementation of physicians (n = 42) to EBGs for VAP prevention. The data indicate that while physicians are generally adhering well to EBGs for VAP prevention, specific practices, particularly those involving protective measures during suctioning and pre-suctioning procedures, require attention to improve compliance. Addressing barriers related to education, potential adverse effects, and a lack of resources could significantly enhance adherence to these guidelines.

**Methods:**

A descriptive correlational design with cross-sectional comparison was used in the study. The population included healthcare professionals in the ICU. Data collection utilized validated questionnaires on knowledge and adherence to VAP prevention guidelines. A total enumeration sampling was done. Data analysis utilized descriptive statistics. Knowledge and compliance scores were converted to levels ranging from poor to excellent based on the percentage of correct answers or compliance incidents. One-way ANOVA and student t-tests were used to analyze knowledge test mean scores.

**Figure:**
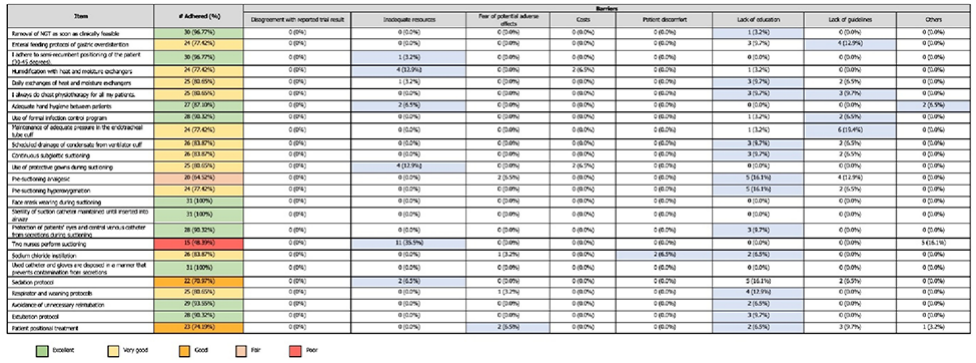
Self-reported adherence and barriers to Implementation of ICU nurses (n = 31) to EBGs for VAP prevention. Adherence to the practice of having two nurses perform suctioning was "Poor" (48.39%), with barriers like disagreement with trial results (35.5%) and other unspecified reasons (16.1%) contributing to the low adherence rate. This reflects a need to address not only knowledge gaps but also the reasons behind nurses' reluctance to follow this guideline. Generally, while nurses showed strong adherence to several key practices, areas like suctioning, analgesic use, and patient positioning could benefit from targeted educational programs and clarification of evidence-based guidelines to improve adherence and overcome barriers.

**Figure:**
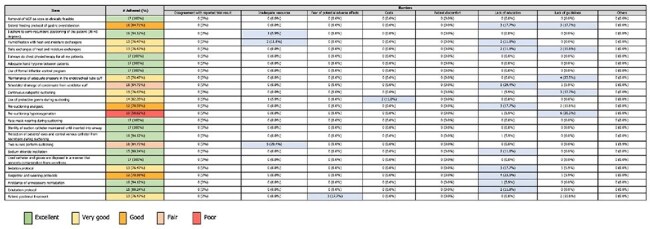
Self-reported adherence and barriers to Implementation of respiratory therapists (n = 17) to EBGs for VAP prevention. For practices such as pre-suctioning analgesic use (70.59%) and respirator and weaning protocols (70.59%), adherence falls into the "Good" category. Respiratory therapists cited educational barriers (17.7%) and lack of guidelines (23.5%) as major factors that affected compliance, indicating gaps in knowledge and available resources.

**Results:**

All groups showed excellent knowledge, with the physicians having the highest level. However, knowledge gaps were identified in areas such as suction systems and airway humidifiers. Physicians reported goof adherence to patient positioning and hand hygiene. On the other hand, nurses had excellent adherence to infection control practices such as maintaining sterility while suctioning and wearing face mask during suctioning. Respiratory therapists showed excellent adherence to chest physiotherapy and patient positioning. Common barriers across all groups included lack of education, inadequate resources, and disagreement with trial results.

**Conclusion:**

The study revealed significant disparities in knowledge of Ventilator-Associated Pneumonia bundles among healthcare professionals in the ICU. The study underscores the need for continuous education and training for all ICU healthcare professionals, with a particular focus on nurses and respiratory therapists, to ensure comprehensive understanding and effective implementation of VAP prevention protocols.

**Disclosures:**

All Authors: No reported disclosures

